# Computational Investigation of the Formation of Peroxide (ROOR) Accretion Products in the OH- and NO_3_-Initiated Oxidation of α-Pinene

**DOI:** 10.1021/acs.jpca.1c08969

**Published:** 2021-12-09

**Authors:** Galib Hasan, Rashid R. Valiev, Vili-Taneli Salo, Theo Kurtén

**Affiliations:** † Department of Chemistry, University of Helsinki, POB 55, Helsinki FIN-00014, Finland; ‡ Institute for Atmospheric and Earth System Research, Faculty of Science, University of Helsinki, Helsinki 00014, Finland; § Research School of Chemistry & Applied Biomedical Sciences, National Research Tomsk Polytechnic University, Lenin Avenue 30, Tomsk 634050, Russia

## Abstract

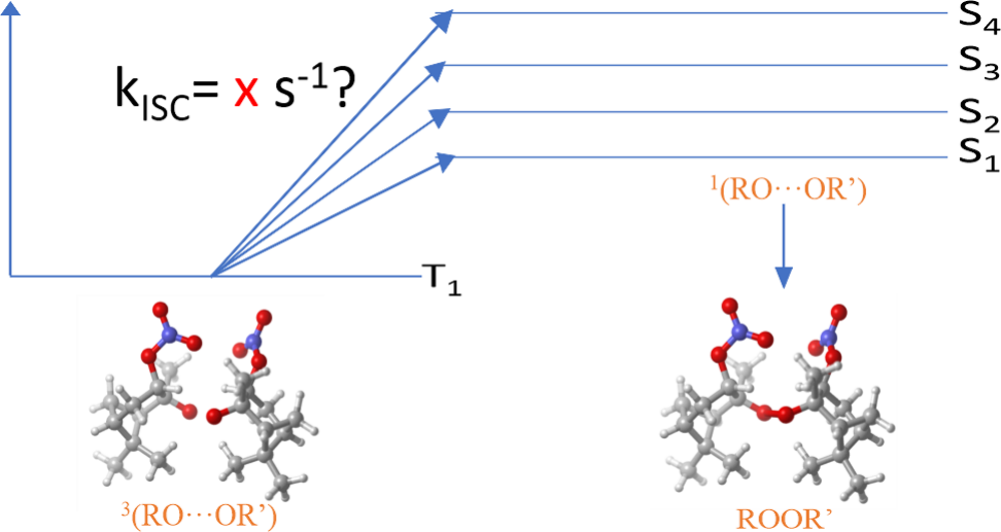

The formation of accretion products (“dimers”) from recombination reactions of peroxyl radicals (RO_2_) is a key step in the gas-phase generation of low-volatility vapors, leading to atmospheric aerosol particles. We have recently demonstrated that this recombination reaction very likely proceeds via an intermediate complex of two alkoxy radicals (RO···OR′) and that the accretion product pathway involves an intersystem crossing (ISC) of this complex from the triplet to the singlet surface. However, ISC rates have hitherto not been computed for large and chemically complex RO···OR′ systems actually relevant to atmospheric aerosol formation. Here, we carry out systematic conformational sampling and ISC rate calculations on ^3^(RO···OR′) clusters formed in the recombination reactions of different diastereomers of the first-generation peroxyl radicals originating in both OH- and NO_3_-initiated reactions of α-pinene, a key biogenic hydrocarbon for atmospheric aerosol formation. While we find large differences between the ISC rates of different diastereomer pairs, all systems have ISC rates of at least 10^6^ s^–1^, and many have rates exceeding 10^10^ s^–1^. Especially the latter value demonstrates that accretion product formation via the suggested pathway is a competitive process also for α-pinene-derived RO_2_ and likely explains the experimentally observed gas-phase formation of C_20_ compounds in α-pinene oxidation.

## Introduction

Organic peroxyl radicals (RO_2_•) are important molecules in the atmosphere because their reactions play a significant role in the formation of low-volatility products, leading to secondary organic aerosol (SOA) particles. Atmospheric aerosols, especially fine <1 μm and ultrafine <100 nm particles, are regarded as one of the key species responsible for air pollution-related mortality.^1^ They also affect the climate by cloud, mist, and fog formation^2^ and contribute to the earth’s energy budget by scattering and absorbing solar radiation and by forming cloud condensation nuclei.^3^ Aerosol-related effects are regarded as one of the least understood components of global radiative forcing.^4^

SOA formation is driven by the oxidation of volatile organic compounds (VOC) such as anthropogenic or biogenic hydrocarbons.^5^ As the direct addition of O_2_ to hydrocarbons is spin-forbidden (at least for ground-state singlet products), this oxidation is initiated by a small number of photochemically generated oxidants: OH, O_3_, Cl, and NO_3_.^6,7^ The details of the oxidation mechanisms depend on the hydrocarbon-oxidant combination, but inevitably involve the formation of peroxyl radicals (RO_2_). In most atmospheric conditions, the main sink of RO_2_ is the reaction with either nitric oxide (NO) or with the hydroperoxyl radical (HO_2_). Self- and cross-reactions of RO_2_ can be major side channels and are especially important for aerosol formation because of their potential to form “dimers”: low-volatility accretion products retaining most or all of the carbon atoms of the original hydrocarbons.

**Scheme 1:**
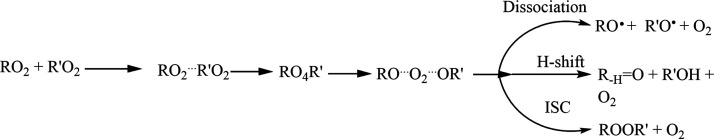
Mechanism for the Cross-Reaction between Two Peroxyl Radicals (RO_2_), and Possible End Products

The molecular-level mechanism of RO_2_ + R′O_2_ reactions was identified as a major open question in atmospheric science already in 2009.^8^ Using multireference quantum chemical calculations,^9,10^ we have recently confirmed the feasibility of the reaction mechanism postulated by Ghigo et al.^11^ and Lee et al.^12^ (see Scheme 1 for illustration). Briefly, all reaction pathways start on an overall singlet potential energy surface and involve at least two intermediates: RO_4_R′ tetroxides as postulated already by the Russell mechanism in 1959^13^ and RO···O_2_···R′O complexes. For the reaction to be thermodynamically feasible, O_2_ must be formed in its triplet ground state. This, in turn, requires that the two alkoxy radicals (RO and R′O) are also coupled as a triplet, preventing immediate recombination because of the Pauli principle. The different reaction channels available to a given RO_2_ + R′O_2_ combination then correspond to different fates of ^3^(RO···OR′) complexes, which are left after the (presumably very weakly bound) ^3^O_2_ has dissociated from the RO···O_2_···R′O system. For example, the dissociation of the cluster leads to RO + R′O products, and intermolecular hydrogen shifts lead to alcohol and carbonyl products. A third possibility is an intersystem crossing (ISC) to the singlet potential energy surface, and subsequent recombination of the alkoxy radicals to form peroxide (ROOR′) products. Our calculations on a variety of relatively simple model systems^9^ demonstrate that all of these channels may have high rate constants, on the order of 10^9^ s^–1^ or more. This suggests that the experimentally observed gas-phase formation of accretion products in various hydrocarbon oxidation systems^14^ may indeed proceed through the ISC of ^3^(RO···OR′) complexes. However, ISC rates have so far not been systematically computed for actual SOA-relevant systems such as monoterpene oxidation products.

**Scheme 2:**
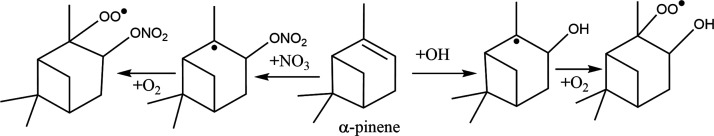
Major Reaction Pathways for the **α**-pinene + NO_3_ and **α**-pinene + OH Systems

Monoterpenes, with elemental composition C_10_H_16_, are biogenic hydrocarbons believed to be important especially for the first steps of SOA formation. The reason for this is that they are large and complex enough to form oxidation products with very low volatilities, while still having high enough emission rates and atmospheric concentrations. α-pinene is one of the most important monoterpenes, accounting for approximately half of all monoterpene emissions.^15^ Reactions between α-pinene and atmospheric oxidants produce a range of products, including multifunctional low-volatility accretion products with up to 20 carbon atoms.^16^ We studied RO_2_ formed in the OH- and NO_3_-initiated oxidation of α-pinene because their structures are unambiguously known and because they have relatively few conformers (compared, for example, to O_3_-derived RO_2_). Scheme 2 depicts the major reaction routes for the oxidation of α-pinene by OH and NO_3_. Basically, the major route involves the addition of OH or NO_3_ to the less highly substituted olefinic carbon atom^7,17^ (H abstraction and/or addition to the other olefinic carbon are possible, but minor, channels). In both cases, the product is an alkylfree radical. O_2_ rapidly adds to this free radical and forms a peroxyl radical RO_2_.^18^

While atmospheric chemistry studies of monoterpenes have traditionally focused mainly on O_3_- and OH-initiated oxidation, recent global modeling studies of organic aerosol^19−21^ suggest that a large fraction of SOA^22,23^ is produced from the oxidation of biogenic organic compounds by the nitrate radical (NO_3_), possibly more than that produced by OH oxidation. Recent studies have further shown that NO_3_ oxidation is not only a night-time process, but happens also during the day.^24,25^ In addition, field analysis of organonitrate diurnal variations demonstrates that NO_3_ oxidation chemistry makes significant contribution to the production of organo-nitrates.^26−28^ Depending on their subsequent photochemistry, organo-nitrates formed by NO_3_-initiated oxidation constitute a large NO_*x*_ reservoir.^29^

In this study, we systematically compute energetics for a large set of ^3^(RO···OR′) clusters corresponding to the relevant RO_2_ + R′O_2_ systems. We consider different stereoisomers of RO derived from the major RO_2_ formed in the OH- and NO_3_-initiated oxidation α-pinene (shown in the right and left sides of Scheme 2, respectively). Because of the presence of two stereocenters, there are four stereoisomers in each system. We denote these as α-pinene, (1) S-alkoxy, R-hydroxy/nitroxy, (2) R-alkoxy, S-hydroxy/nitroxy, (3) S-alkoxy, S-hydroxy/nitroxy, and (4) R-alkoxy, R-hydroxy/nitroxy. In our previous work, we developed a configurational sampling approach for ^3^(RO···OR′) clusters.^30^ We use the same approach here to search for the global minimum-energy conformer for each cluster type, considering the homodimers [i.e., ^3^(RO···OR′) clusters, where RO and RO′ are the same species] of all monomer stereoisomers. Dimers consisting of different monomer stereoisomers were not considered because of computational reasons (i.e., the large number of such systems). We are not aware of any reported stereoselectivity of the reactions shown in Scheme 2 (or of any of the competing RO_2_ sink reactions), and thus, the collision of each combination of two stereoisomers could be presumed equally likely. The set of ^3^(RO···OR′) clusters studied here should thus be considered a representative subset. However, as discussed below, our results on the alkoxy-nitroxy systems tentatively suggest that the formation of one of the four stereoisomers may be energetically unfavorable compared to the others.

For the studied ^3^(RO···OR′) clusters, we then calculated the ISC rate constant using state-of-the-art multireference methods. We did not study the H-shift channel in this work because there are no H atoms to abstract on the tertiary α-oxyl carbon. The conventional “alcohol + carbonyl” channel is thus not possible for the studied RO_2_/RO systems. Hypothetical H-shifts from other C atoms are likely to have high barriers because of a combination of steric strain and the formation of diradical products.

We note that while the overall self-reaction rate of tertiary peroxy radicals is often low, OH-substituted tertiary peroxy radicals can have self-reaction rate coefficients on the order of 10^–14^ cm^3^ mol^–1^ s^–1^.^7^ If accretion product (ROOR′) formation is the dominant channel for these reactions, they may be atmospherically relevant despite representing a relatively minor RO_2_ sink compared to reactions with NO or HO_2_.

## Theory and Methods

### Conformational Sampling of the Alkoxy Radical Monomers

The systematic conformer search algorithm in Spartan version 16 was used for generating all the conformers of the alkoxy monomers (RO•).^31^ In this approach, every nonterminal bond is rotated by 180 degrees for sp^2^-hybridized atoms and 120 degrees for sp^3^-hybridized atoms. Possible ring flipping was also considered during this conformer searching. A molecular mechanics force-field (MMFF) optimization was then performed to find representative sets of all the local minima conformers on the potential energy surfaces (PES). We found a maximum of three conformers for each alkoxy radical stereoisomer because torsional rotations are limited by the intact bicyclic α-pinene skeleton (see Scheme 2). We used the keyword (“ffhint = O_*x*_ ∼ ∼6,” denoting generic divalent O) to avoid the treatment of alkoxy radical oxygens as anions.^32^ (We note that this issue has been resolved in newer versions of Spartan, from 18 onward). For the S-alkoxy,*R*-nitroxy system, conformational sampling with Spartan 16 yielded only one monomer conformer. This structure was therefore resampled using Spartan 20, leading to three conformers, which were then used in the subsequent sampling of ^3^(RO···OR′) clusters.

### Systematic Conformational Sampling of ^3^(RO···OR′) Clusters

The conformational sampling of ^3^(RO···OR′) clusters involved several steps. First, thousands of initial conformers were generated using the artificial bee colony (ABC) algorithm, which performs rigid-body molecular dynamics.^33,34^ The ABC algorithm requires monomer structures, as well as Lennard-Jones parameters and partial charges for all atoms in the monomers. Structures and partial charges were obtained from optimizations and natural bonding orbital (NBO) calculations at the ωB97X-D/6-31++G** level of theory using Gaussian16 RevB.01.^35^ The Lennard-Jones parameters were collected from the CHARMM force-field database. We initially generated 3000 cluster conformers for every combination of monomer conformers. As described above, each monomer had a maximum of three conformers, leading to six distinct combinations of two monomer conformers. Thus, a total of up to 18,000 conformers were generated for each of the eight studied stereoisomers. Semiempirical optimization was then carried out using the XTB program and the GFN-*x*TB (Geometry, Frequency, Noncovalent, eXtended Tight Binding) level of theory^36^ for all the conformers generated using the ABC algorithm. At this stage, various unwanted reactions, typically combinations of H-shifts and C–C bond scissions, took place because of a combination of strained input geometries and (likely artificially) low reaction barriers at the GFN-*x*TB level. Section S1 of the Supporting Information shows some examples of unwanted reactions at the XTB level. We tested different keyword settings in the XTB program to get rid of this problem. We found that performing the semiempirical optimizations with loose optimization criteria helped get rid of most of the unwanted reactions at least for the clusters studied here. Conformers within 10 kcal/mol of the lowest-energy structure for each system were then selected for density functional level (DFT) calculations. Because of the large size of the α-pinene systems, computational costs prevented us from carrying out calculations at the same level as in our previous studies (coupled cluster singles, doubles and perturbative triples [CCSD(T)] energies on ωB97X-D/aug-cc-pVTZ structures). Therefore, DFT optimizations were performed at the ωB97X-D/6-31++G** level of theory^37,38^ using Gaussian16 RevB.01.^39^ Electronic energies and dipole moments were then collected from all conformers, and duplicate structures (with identical energies and dipole moments) were eliminated. Finally, ωB97X-D/6-31++G** frequency calculations were performed on conformers within 5 kcal/mol of the lowest-energy structure for each system. We note that while binding energetics computed at this level should be considered qualitative, the actual cluster geometries used for the ISC rate calculations are likely much less sensitive to the size of the basis set. The ISC rates presented here should thus be comparable to our previous studies.

## ISC Rate Calculation

**Figure 1:**
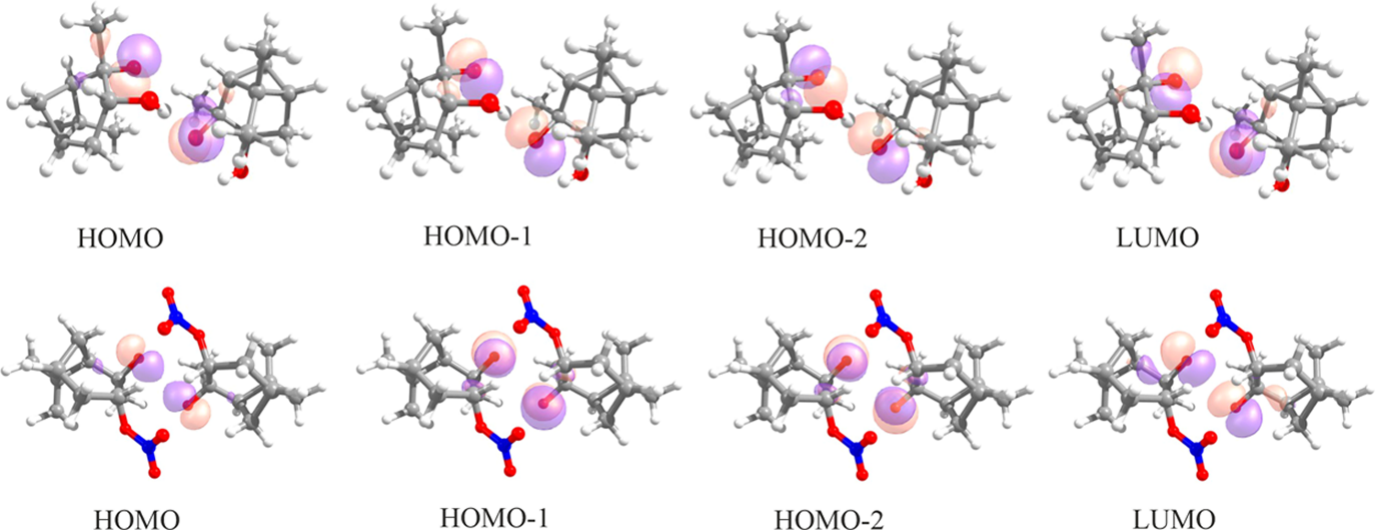
Orbitals included in the (6,4) active space for α-pinene, (*S*-alkoxy,*R*-hydroxy)_2_ and α-pinene, (*R*-alkoxy,*R*-nitroxy)_2_. HOMO and LUMO refer to the highest occupied and lowest unoccupied molecular orbitals, respectively. Color coding: gray = C, red = O, white = H, and blue = N.

^3^(RO···OR′) clusters can undergo ISC to the singlet surface, allowing for (presumably near-instantaneous) recombination to covalently bound ROOR accretion products. The details of our ISC rate calculations are explained in our previous work.^9,10^ In brief, the global minimum conformers from the DFT calculations described above, as well as one other conformer per system (the lowest-energy conformer that had a clearly different bonding pattern compared to the global minimum), were selected, and the energies of the lowest four singlet and triplet states were computed at the XMC-QDPT2/6-311++G** level of theory using Firefly, version 8.2.0.^40^ The ISC rate coefficient, *k*_ISC_ (in units of s^–1^), was then obtained using the following formula:
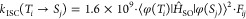
1where ⟨φ(*T_i_*) | *Ĥ*_SO_ | φ(*S_j_*)⟩ is the spin-orbit coupling matrix element (SOCME) in cm^–1^, and *F_ij_* is Franck–Condon’s factor (which depends on the energy gap between the states). We tested several different active spaces to check which sets of orbitals contribute to the state averaging. We found that the (6,4) active space (six electrons in four orbitals) represents a good compromise between the computational cost and accuracy and is sufficient to describe the states of interest. The details of the active space selection followed the philosophy described in our previous work.^10^ The selected molecular orbitals, mainly formed from *p*-atomic orbitals of the radical oxygen atoms, are those that give the largest contributions (configuration interaction weights of more than 0.2) to the relevant low-lying electronic states (T_1_...T_4_ and S_1_...S_4_). Figure 1 shows the orbitals included in the active space for two of the eight studied systems (one alkoxy-hydroxy and one alkoxy-nitroxy radical pair). The orbitals for other stereoisomer pairs look similar. ISC rates were computed for transitions from the triplet ground state T_1_ to the four lowest singlet states (S_1_...S_4_).

The matrix element of the spin-orbit coupling interaction between triplet T_1_-T_4_ and singlet states S_1_-S_4_ was calculated at the CASSCF(6,4)/ 6-311++G** level of theory, but using the XMC-QDPT2/6-311++G** energies, with the general atomic and molecular electronic structure system (GAMESS-US) program.^41^

## Results and Discussion

**Figure 2:**
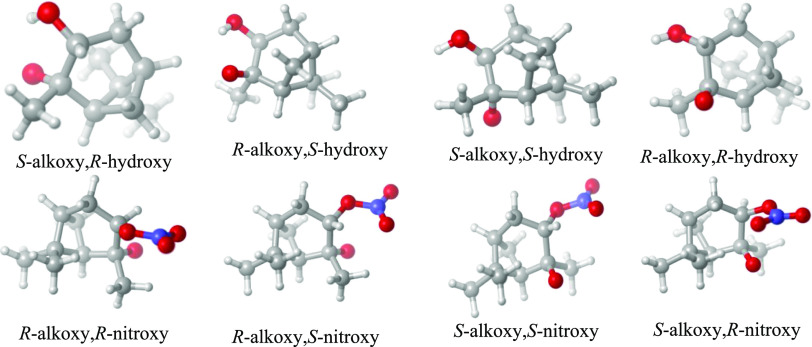
Optimized lowest-energy structures at the ωB97X-D/6-31++G** level of theory of different stereoisomers of the hydroxy-alkoxy and nitroxy-alkoxy radicals formed in the oxidation of α-pinene by OH and NO_3_, assuming initial radical addition to the secondary carbon atom (as depicted in Scheme 2, followed by the loss of one oxygen from the peroxy radicals to form alkoxy radicals). Color coding: gray = C, white = H, red = O, and blue = N.

**Figure 3:**
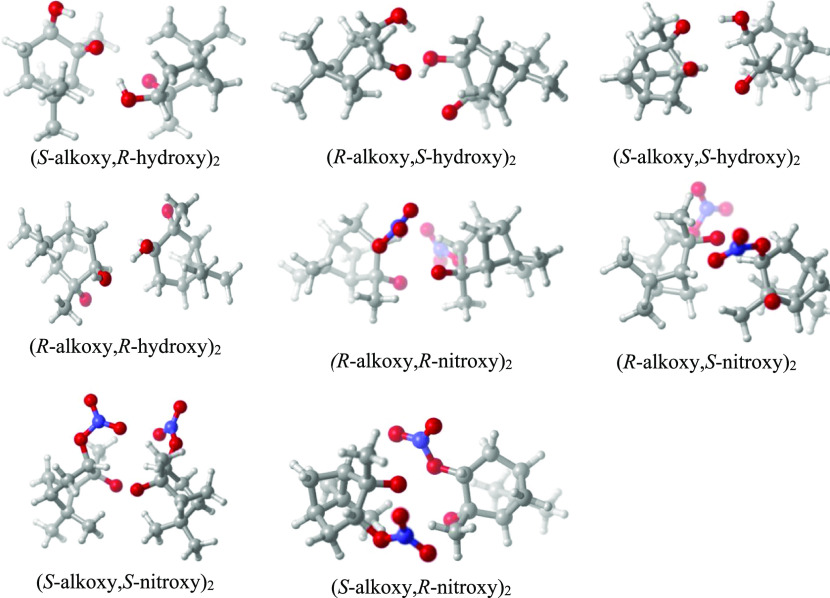
Optimized lowest-energy structures of the ^3^(RO···OR′) clusters studied in this work at the ωB97X-D/6-31++G** level of theory. Color coding: gray = C, white = H, red = O, and blue = N.

Minimum-energy conformers of the RO monomers are shown in Figure 2, while the minimum-energy conformers of the ^3^(RO···OR′) clusters are given in Figure 3. (Structures and relative energies of local minima are given in Section S4 of the Supporting Information). The relative binding energies of the ^3^(RO···OR′) clusters (expressed in terms of the energies and Gibbs free energies of the ^3^(RO···OR′) → RO + R′O reaction) are given in Table 1. Consistent with the DFT results on smaller functionalized RO in our previous study,^9^ the electronic energies of the dissociation reaction mostly vary between about 9 and 12 kcal/mol, while the corresponding Gibbs free energies vary between about 1 and –3 kcal/mol. These values are typical for fairly weakly bonded clusters in the atmosphere. The α-pinene,(*S*-alkoxy,*R*-nitroxy)_2_ cluster is an outlier, with considerably stronger bonding than any of the other clusters. As seen from Figure 3, this cluster has a direct interaction between the alkoxy radical on one monomer and the nitroxy group of the other monomer. Most other alkoxy-nitroxy clusters in this study, as well as the simpler alkoxy-nitroxy clusters in our previous study,^9^ had global minimum structures (at the at ωB97X-D level) characterized by nitroxy-nitroxy interactions. The α-pinene,(*R*-alkoxy,*S*-nitroxy)_2_ cluster also contains an alkoxy-nitroxy interaction, but has a much lower binding energy. Upon closer inspection, the anomalous binding energy of the α-pinene,(*S*-alkoxy,*R*-nitroxy)_2_ system is driven mainly by differences in monomer energies: the α-pinene, *S*-alkoxy,*R*-nitroxy radical monomer is between 1.6 and 4.0 kcal/mol higher in absolute energy than the other three alkoxy-nitroxy radicals, presumably because of less favorable interactions between the nitroxy group and the rest of the molecule in the monomer. As the monomer energy is multiplied by two in the binding energy calculation, this alone leads to a difference between 3.2 and 8.0 kcal/mol in the binding energies in favor of the α-pinene, (*S*-alkoxy,*R*-nitroxy)_2_ system. In contrast, the differences in absolute energies of the four different alkoxy-nitroxy clusters shown in Figure 3 are less than 5 kcal/mol. To ensure that the presented α-pinene, (*S*-alkoxy,*R*-nitroxy)_2_ structure is not a computational artifact, we have recomputed its binding energy with the same basis set, but using four different functionals (PW6B95D3, M05-2X, M06-2X, and LC-wPBE, using GD3 empirical dispersion), and found similar results, as obtained at the ωB97X-D/6-31++G** level. We also manually constructed clusters with similar bonding patterns for the other three systems in order to rule out the possibility that the “anomalous” bonding pattern is actually the correct one but was simply missed for the other three cases because of the limitations of our conformational sampling algorithm. However, these did not lead to lower-energy clusters than those already discovered in the sampling. Based on these test calculations, we tentatively conclude that the anomalously strong binding of the α-pinene, (*S*-alkoxy,*R*-nitroxy)_2_ system represents an example of genuinely strong stereoselectivity in cluster formation. However, we note that the high (unfavorable) energy of the corresponding monomer, assuming a similar pattern, also found for the parent peroxy-nitroxy radicals, may also cause stereoselectivity in the formation reaction: the *S*-peroxy,*R*-nitroxy stereoisomer may have a much lower yield than the others.

**Table 1:** Electronic Energies (in kcal/mol) and Gibbs Free Energies (in kcal/mol at 298 K and 1 atm Reference Pressure) for the ^3^(RO···OR′) → RO + R′O Reaction Computed at ωB97X-D/6-31++G** Level of Theory

^3^(RO···OR′) cluster	Δ*E* in kcal/mol	Δ*G* in kcal/mol	O···O radical distance in Å
α-pinene, (*S*-alkoxy,*R*-hydroxy)_2_	+9.14	–3.12	3.31
α-pinene, (*R*-alkoxy,*S*-hydroxy)_2_	+9.10	–2.96	3.35
α-pinene, (*S*-alkoxy,*S*-hydroxy)_2_	+9.76	–2.22	4.13
α-pinene, (*R*-alkoxy,*R*-hydroxy)_2_	+11.96	+0.97	3.50
α-pinene, (*R*-alkoxy,*R*-nitroxy)_2_	+10.71	–1.89	3.19
α-pinene, (*R*-alkoxy,*S*-nitroxy)_2_	+10.18	–1.63	5.49
α-pinene, (*S*-alkoxy,*S*-nitroxy)_2_	+10.83	–2.61	3.48
α-pinene, ( *S*-alkoxy,*R*-nitroxy)_2_	+18.19	+6.34	4.32

Even if the *S*-alkoxy,*R*-nitroxy system is excluded, the nitroxy-alkoxy radical dimers are generally somewhat more strongly bound than their hydroxy-alkoxy counterparts, which is surprising as the latter have H-bonds while the former do not. This is likely explained by the presence of intramolecular H-bonds in the hydroxy-alkoxy radical monomers. These act to decrease the energies (and free energies) of the monomers, leading to relatively weaker binding of the clusters. The distance between the two radical oxygen atoms did not correlate significantly with the cluster binding energy, likely because even the shortest distances were above 3 Å (implying little interaction).

The overall ISC rate constants for our studied systems (corresponding to a sum of the four-individual computed ISC rates) are given in Table 2. (Data for local minima are given in Section S2 of the Supporting Information.) In our previous work comparing a series of relatively simple functionalized RO,^9,10^ we observed that the ISC rates strongly depended on the conformation of the ^3^(RO···OR′) clusters and displayed extreme stereoselectivity for the two systems included in the study that possessed stereocenters: *R,S*-BuOH-O···O-BuOH and *R,S*-PrNO_3_-O···O-PrNO_3_. The present systems also display substantial stereoselectivity: the ISC rates for the global minimum conformers of different stereoisomer pairs of hydroxy-alkoxy and nitroxy-alkoxy triplet clusters vary by almost three and over four orders of magnitude, respectively. The variation between different conformers of the same system is also large, up to four orders of magnitude for the studied pairs of global and local minima. Notably, for some of the systems where the global minimum conformer had a relatively low ISC rate, the local minimum conformer (with a different binding pattern) had a substantially higher rate or vice versa. All computed ISC rates are in any case fairly fast, exceeding 10^7^ s^–1^ for all but three of the 16 cases (when including the local minima) and 10^8^ s^–1^ for over half the cases.

**Table 2:** ISC Rate Constants for The Studied Systems at 298 K, Based on Energies of Triplet and Singlet States Computed Using XMC-QDPT2(10,8)/6-311++G** and Matrix Elements of the Spin-Orbit Coupling Interaction between T_1_-T_4_ and S_1_-S_4_ States Computed at the CASSCF(6,4)/6-31++G** Level

^3^(RO···OR′) cluster	Σ*k*_ISC_ (s^–1^)
α-pinene, (*S*-alkoxy,*R*-hydroxy)_2_	1.18 × 10^10^
α-pinene, (*R*-alkoxy,*S*-hydroxy)_2_	5.68 × 10^7^
α-pinene, (*S*-alkoxy,*S*-hydroxy)_2_	9.43 × 10^9^
α-pinene, (*R*-alkoxy,*R*-hydroxy)_2_	1.51 × 10^10^
α-pinene, (*R*-alkoxy,*R*-nitroxy)_2_	1.62 × 10^10^
α-pinene, (*R-*alkoxy,*S*-nitroxy)_2_	1.02 × 10^6^
α-pinene, (*S*-alkoxy,*S*-nitroxy)_2_	5.68 × 10^10^
α-pinene, ( *S*-alkoxy,*R*-nitroxy)_2_	2.06 × 10^8^

Section S2 of the Supporting Information shows the relative energies of all considered electronic states, as well as the SOCME values, and the ISC rates for individual transitions. The overall ISC rate of the global minimum conformers is dominated by the ISC between T_1_ and S_1_ for all the studied systems except for two: (*R-*alkoxy,*S*-nitroxy)_2_ and (*S*-alkoxy,*S*-nitroxy)_2_. In the former case, the SOCME between T_1_ and S_1_ is zero, presumably due to the very large distance (5.49 Å) between the radical centers,^42^ leading to a zero ISC rate between these states and a low overall ISC rate. In the latter case, the SOCME between the T_1_ and S_1_ states is small (0.17 cm^–1^), leading to a modest ISC rate for this transition (4.62 × 10^7^ s^–1^) despite the fact that the energy gap is negative (i.e., the S_1_ state is lower than T_1_ for this system, unlike the other seven). However, the overall ISC rate for this system is still high because of the exceptionally high ISC rates between T_1_ and S_2_ as well as S_3_.

The variation in the ISC rate between T_1_ and S_1_, which determines the overall ISC rate for the remaining six systems, is driven almost exclusively by the variations in the SOCME, as the corresponding energy gaps are very low (less than 80 cm^–1^). The low energy gap between T_1_ and S_1_ is consistent with the large distance between the radical centers (Table 2) and the consequent lack of strong interactions between them. The variations in the SOCME between T_1_ and S_1_ can in turn be related to the orientation of the C–O bonds relative to each other. The wavefunctions of the T_1_ and S_1_ states have almost the same electronic configuration, with contributions mainly from the 2p atomic orbitals (AO).^42^ Thus, the SOCME depends on the overlap between 2p-AOs, belonging to the radical oxygen atoms of two radicals, which in turn depends on their relative orientation.^42,43^ In the future, for example, machine-learning approaches could be used to cost-effectively predict SOCME and thus ISC rates for ^3^(RO···OR′) systems based on the relative orientation of the alkoxy groups.

We further tested the validity of our hypothesis that an ISC in the ^3^(RO···OR′) clusters will inevitably lead to prompt ^1^ROOR′ formation. This was done by simply reoptimizing the obtained ^3^(RO···OR′) minimum-energy structures (shown in Figure 3) on the singlet potential energy surface using the same DFT method (ωB97X-D/6-31++G**). The results of the singlet optimization are shown in Section S3 of the Supporting Information. For five of our eight systems, the optimization yielded the expected formation of ^1^ROOR′ structures. For the remaining three cases, all of which corresponded to clusters in which the radical centers were relatively far from each other, various other reactions occurred instead, including intramolecular H-shifts and C–C scissions. We caution that while both C–C scissions and H-shifts are well-documented reaction classes of alkoxy radicals, such reactions tend to have at least moderate energy barriers and would not be expected to occur in simple optimizations (energy minimizations). These reactions may thus be artifacts of the relatively low-level optimization method, which is unable to properly treat the ^1^(RO···OR′) starting point as an open-shell singlet. In any case, these test calculations suggest that ^1^ROOR′ formation is indeed likely to be the major, although not necessarily the exclusive, end result of an ISC in our ^3^(RO···OR′) systems.

## Conclusions

Self- and cross-reactions of peroxyl radicals play important roles in both atmospheric and combustion chemistry. For simple peroxyl radicals in the gas phase, the main channels of these reactions correspond to the formation of either two alkoxy radicals or carbonyl and alcohol products. For more complex reactants, experimental studies indicate that also the formation of ROOR′ accretion products is a competitive pathway. Gas-phase accretion product formation provides an efficient mechanism for atmospheric aerosol formation, as it dramatically lowers the volatility of the participating compounds in a single step. Computational studies by us and others suggest that ROOR′ formation is preceded by ISC in weakly bound ^3^(RO···OR′) complexes formed as an intermediate step in all peroxyl radical self- and cross-reactions. However, actual ISC rates have never been computed for large ^3^(RO···OR′) systems corresponding, for example, to monoterpene oxidation products. In this study, we first determined the structures and binding energies of ^3^(RO···OR′) complexes formed in the self-reactions of a set of RO_2_ stereoisomers produced in the OH- and NO_3_-initiated oxidation of α-pinene. The overall binding energies were fairly weak (around 10 kcal/mol in electronic energy for all but one system) despite the presence of H-bonds in the OH-oxidized systems, implying rapid dissociation rates for the ^3^(RO···OR′) complexes. The computed ISC rates were also high, exceeding 10^6^ s^–1^ for all systems and 10^10^ s^–1^ for some systems. At least the fastest rates should be competitive in atmospheric conditions, confirming the hypothesis that the proposed mechanism can explain accretion product formation observed in α-pinene oxidation experiments. The significant variation of the calculated ISC rates between stereoisomers further implies that accretion product formation might be stereoselective: only some of the RO_2_ diastereomers formed in monoterpene oxidation may be able to form ROOR′ effectively. However, the large variation in ISC rates between low-energy conformers of the same stereoisomers is likely to decrease the stereoselectivity of ROOR′ formation.

## Supporting Information Available

Data on local minimum conformers, unwanted reactions occurring in the conformational sampling, results of optimizations on the singlet surface, and detailed ISC rate data for all the systems studied here (PDF)
Quantum chemical output files (ZIP)
