# Supplementary material for: Computational Investigation of the Formation of Peroxide (ROOR) Accretion Products in the OH- and NO3-Initiated Oxidation of α-Pinene
Source: J Phys Chem A. 2021 Dec 9;125(50):10632–9. doi: 10.1021/acs.jpca.1c08969 (PMC8713291; doi:10.1021/acs.jpca.1c08969)
Supplement: Supplementary file 1 — jp1c08969_si_001.pdf [file jp1c08969_si_001.pdf]

Supporting Information

Computational Investigation of the Formation of Peroxide (ROOR) Accretion Products in the OH- and NO_3 – Initiated Oxidation of α-pinene

Galib Hasan[a,b*], Rashid R. Valiev[a,b,c], Vili-Taneli Salo[a,b], Theo Kurten[a,b*]

[a]Department of Chemistry, University of Helsinki, POB 55, FIN-00014 Helsinki, Finland

[b]Institute for Atmospheric and Earth System Research, Faculty of Science, University of Helsinki, Helsinki 00014, Finland

[c]Research School of Chemistry & Applied Biomedical Sciences, National Research Tomsk Polytechnic University, Lenin Avenue 30, Tomsk 634050, Russia

Contents

Section S1. Examples of unwanted reactions occurring in the configurational sampling at the XTB level. (Figure S1)

Section S2. The SOCME (cm^{-1}), Energy Gap (cm^{-1}), and k_{ISC} (s^{-1}) computed for all (RO···OR') clusters (global minimum and one representative local minimum conformer for each system). (Tables S1…S16)

Section S3. Results of optimization of the (RO···OR') complexes on the singlet surface ^{1}(RO···OR'). (Figure S2)

Section S4. Optimized structures of the representative local minima conformer used for calculating the ISC rates in section S2 (even-numbered tables). (Figure S3)

Section S1. Examples of unwanted reactions occurring in the configurational sampling at the XTB level.

Figure S1. Figure of different unwanted reactions that happened during XTB optimizations. Color coding: brown=C, white=H, red=O.

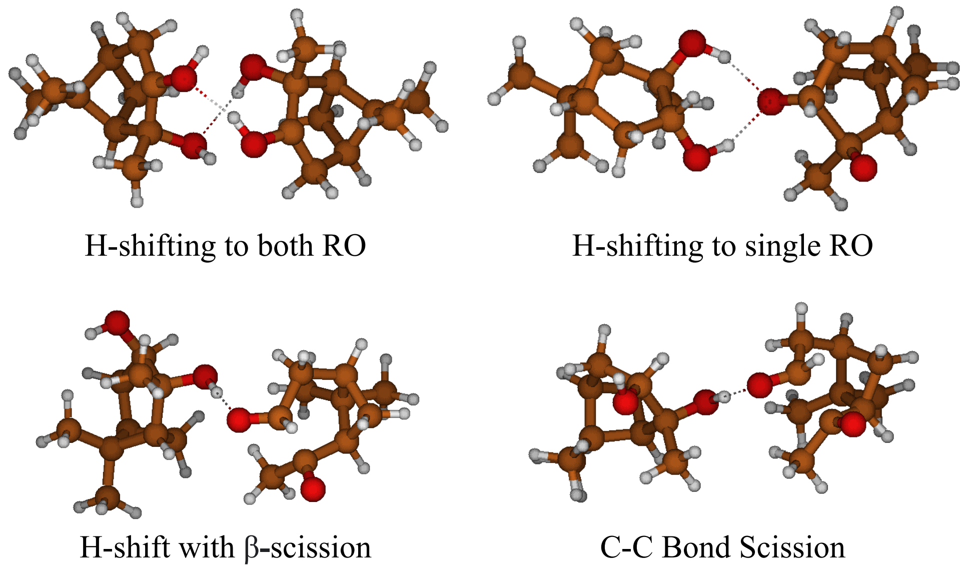

Section S2. SOCME (cm^{-1}), Energy Gap (cm^{-1}), and k_{ISC} (s^{-1}) computed for all (RO···OR') clusters (global minimum and one representative local minima conformer). See Section S4 for structures and relative energies of local minima.

Table S1: The SOCME (cm^{-1}), Energy Gap (cm^{-1}), and k_{ISC} (s^{-1}) computed for α-pinene, (*S*-alkoxy,*R*-hydroxy)$_2$ cluster (global minima).

Transition	SOCME	Energy Gap	k_{ISC} (s^{-1})
$T_1 \rightarrow S_1$	2.9	26.4	1.2×10^{10}
$T_1 \rightarrow S_2$	98.51	3146.3	2.0×10^{5}
$T_1 \rightarrow S_3$	101.41	5150.0	2.3×10^{0}
$T_1 \rightarrow S_4$	1.42	8376.0	5.0×10^{-12}
		Total rate	1.2×10^{10}

Table S2: The SOCME (cm^{-1}), Energy Gap (cm^{-1}), and k_{ISC} (s^{-1}) computed for α-pinene, (*S*-alkoxy,*R*-hydroxy)$_2$ cluster (local minima).

Transition	SOCME	Energy Gap	k_{ISC} (s^{-1})
$T_1 \rightarrow S_1$	0.2	25.9	5.7×10^{7}
$T_1 \rightarrow S_2$	104.23	3147.0	2.2×10^{5}
$T_1 \rightarrow S_3$	105.71	5149.6	2.6×10^{0}
$T_1 \rightarrow S_4$	0.45	8377.6	4.9×10^{-13}
		Total rate	5.7×10^{7}

Table S3: The SOCME (cm^{-1}), Energy Gap (cm^{-1}), and k_{ISC} (s^{-1}) computed for α-pinene, (*R*-alkoxy,*S*-hydroxy)$_2$ cluster (global minima).

Transition	SOCME	Energy Gap	k_{ISC} (s^{-1})
$T_1 \rightarrow S_1$	0.17	10.8	4.1×10^7
$T_1 \rightarrow S_2$	104.06	2378.0	1.7×10^7
$T_1 \rightarrow S_3$	103.98	3788.4	5.6×10^3
$T_1 \rightarrow S_4$	1.03	6168.7	7.4×10^{-7}
		Total rate	5.7×10^7

Table S4: The SOCME (cm^{-1}), Energy Gap (cm^{-1}), and k_{ISC} (s^{-1}) computed for α-pinene, (*R*-alkoxy,*S*-hydroxy)$_2$ cluster (local minima).

Transition	SOCME	Energy Gap	k_{ISC} (s^{-1})
$T_1 \rightarrow S_1$	0.03	15.3	1.3×10^6
$T_1 \rightarrow S_2$	102.5	3489.3	3.0×10^4
$T_1 \rightarrow S_3$	104.96	3500.4	3.0×10^4
$T_1 \rightarrow S_4$	0.77	6982.0	4.0×10^{-9}
		Total rate	1.4×10^6

Table S5: The SOCME (cm^{-1}), Energy Gap (cm^{-1}), and k_{ISC} (s^{-1}) computed for α-pinene, (*S*-alkoxy,*S*-hydroxy)$_2$ (global minima)

Transition	SOCME	Energy Gap	k_{ISC} (s^{-1})
$T_1 \rightarrow S_1$	2.5	21.4	9.0×10^9
$T_1 \rightarrow S_2$	88.85	1784.0	3.7×10^8
$T_1 \rightarrow S_3$	98.36	2237.5	3.4×10^7
$T_1 \rightarrow S_4$	1.83	3950.7	7.0×10^{-1}
		Total rate	9.4×10^9

Table S6: The SOCME (cm^{-1}), Energy Gap (cm^{-1}), and k_{ISC} (s^{-1}) computed for α-pinene, (*S*-alkoxy,*S*-hydroxy)$_2$ (local minima)

Transition	SOCME	Energy Gap	k_{ISC} (s^{-1})
$T_1 \rightarrow S_1$	0.3	20.9	1.3×10^8
$T_1 \rightarrow S_2$	104.29	1786.8	5.0×10^8
$T_1 \rightarrow S_3$	105.64	2238.8	4.0×10^7
$T_1 \rightarrow S_4$	3.03	3955.2	1.9×10^0
		Total rate	6.7×10^8

Table S7: The SOCME (cm^{-1}), Energy Gap (cm^{-1}), and k_{ISC} (s^{-1}) computed for α-pinene, (*R*-alkoxy,*R*-hydroxy)$_2$ cluster (global minima)

Transition	SOCME	Energy Gap	k_{ISC} (s^{-1})
$T_1 \rightarrow S_1$	3.06	15.0	1.5×10^{10}
$T_1 \rightarrow S_2$	67.86	1860.0	1.4×10^{8}
$T_1 \rightarrow S_3$	24.7	2707.2	1.5×10^{5}
$T_1 \rightarrow S_4$	69.54	4622.5	2.2×10^{1}
		Total rate	1.5×10^{10}

Table S8: The SOCME (cm^{-1}), Energy Gap (cm^{-1}), and k_{ISC} (s^{-1}) computed for α-pinene, (*R*-alkoxy,*R*-hydroxy)$_2$ cluster (local minima)

Transition	SOCME	Energy Gap	k_{ISC} (s^{-1})
$T_1 \rightarrow S_1$	0.44	14.0	2.9×10^{8}
$T_1 \rightarrow S_2$	105.42	1857.8	3.4×10^{8}
$T_1 \rightarrow S_3$	104.7	2719.7	2.5×10^{6}
$T_1 \rightarrow S_4$	2.84	4590.0	4.4×10^{-2}
		Total rate	6.3×10^{8}

Table S9: The SOCME (cm^{-1}), Energy Gap (cm^{-1}), and k_{ISC} (s^{-1}) computed for α-pinene, (*R*-alkoxy,*S*-nitroxy)$_2$ cluster (global minima).

Transition	SOCME	Energy Gap	k_{ISC} (s^{-1})
$T_1 \rightarrow S_1$	0.0	163.8	0
$T_1 \rightarrow S_2$	105.7	2800.4	1.1×10^{6}
$T_1 \rightarrow S_3$	105.4	3000.5	5.0×10^{5}
$T_1 \rightarrow S_4$	0.6	6350.0	3.4×10^{-9}
		Total rate	1.0×10^{6}

Table S10: The SOCME (cm^{-1}), Energy Gap (cm^{-1}), and k_{ISC} (s^{-1}) computed for α-pinene, (*R*-alkoxy,*S*-nitroxy)$_2$ cluster (local minima).

Transition	SOCME	Energy Gap	k_{ISC} (s^{-1})
$T_1 \rightarrow S_1$	3.81	78.4	1.6×10^{10}
$T_1 \rightarrow S_2$	69.47	2165.6	2.6×10^{7}
$T_1 \rightarrow S_3$	133.92	2411.1	2.4×10^{7}
$T_1 \rightarrow S_4$	3.47	4587.4	6.7×10^{-02}
		Total rate	1.6×10^{10}

Table S11: The SOCME (cm^{-1}), Energy Gap (cm^{-1}), and k_{ISC} (s^{-1}) computed for α-pinene, (*R*-alkoxy,*R*-nitroxy)$_2$ cluster (global minima).

Transition	SOCME	Energy Gap	k_{ISC} (s^{-1})
$T_1 \to S_1$	3.84	78.0	1.6×10^{10}
$T_1 \to S_2$	70.27	2161.1	2.6×10^{6}
$T_1 \to S_3$	133.49	2405.6	3.0×10^{7}
$T_1 \to S_4$	3.49	4575.0	3.7×10^{-2}
		Total rate	1.6×10^{10}

Table S12: The SOCME (cm^{-1}), Energy Gap (cm^{-1}), and k_{ISC} (s^{-1}) computed for α-pinene, (*R*-alkoxy,*R*-nitroxy)$_2$ cluster (local minima).

Transition	SOC	Energy Gap	k_{ISC} (s^{-1})
$T_1 \to S_1$	0.09	13.8	1.2×10^{7}
$T_1 \to S_2$	105.38	2730.3	2.4×10^{6}
$T_1 \to S_3$	105.83	2864.1	5.6×10^{5}
$T_1 \to S_4$	0.58	5576.3	1.1×10^{-6}
		Total rate	1.5×10^{7}

Table S13 The SOCME (cm^{-1}), Energy Gap (cm^{-1}), and k_{ISC} (s^{-1}) computed for α-pinene, (*S*-alkoxy,*S*-nitroxy)$_2$ cluster (global minima).

Transition	SOCME	Energy Gap	k_{ISC} (s^{-1})
$T_1 \to S_1$	0.17	-2072.0	4.6×10^{7}
$T_1 \to S_2$	85.15	1000.4	3.1×10^{10}
$T_1 \to S_3$	123.18	1200.3	2.0×10^{10}
$T_1 \to S_4$	1.23	4000.5	0.2×10^{-3}
		Total rate	5.7×10^{10}

Table S14: The SOCME (cm^{-1}), Energy Gap (cm^{-1}), and k_{ISC} (s^{-1}) computed for α-pinene, (*S*-alkoxy,*S*-nitroxy)$_2$ cluster (local minima).

Transition	SOC	Energy Gap	k_{ISC} (s^{-1})
$T_1 \to S_1$	0.09	2.0	1.3×10^{7}
$T_1 \to S_2$	19.44	3149.0	7.5×10^{3}
$T_1 \to S_3$	148.56	3173.7	3.8×10^{5}
$T_1 \to S_4$	0.34	6420.5	1.9×10^{-8}
		Total rate	1.3×10^{7}

Table S15: The SOCME (cm^{-1}), Energy Gap (cm^{-1}), and k_{ISC} (s^{-1}) computed for α-pinene, (*S*-alkoxy,*R*-nitroxy)$_2$ cluster (global minima).

Transition	SOCME	Energy Gap	k_{ISC} (s^{-1})
$T_1 \rightarrow S_1$	0.4	2.0	2.1×10^8
$T_1 \rightarrow S_2$	81.0	3050.6	2.3×10^5
$T_1 \rightarrow S_3$	73.1	3832.6	2.2×10^3
$T_1 \rightarrow S_4$	1.1	6886.4	1.3×10^{-8}
		Total rate	2.1×10^8

Table S16: The SOCME (cm^{-1}), Energy Gap (cm^{-1}), and k_{ISC} (s^{-1}) computed for α-pinene, (*S*-alkoxy,*R*-nitroxy)$_2$ cluster (local minima).

Transition	SOCME	Energy Gap	k_{ISC} (s^{-1})
$T_1 \rightarrow S_1$	0	5.1	0.0×10^0
$T_1 \rightarrow S_2$	105.47	3254.1	1.2×10^5
$T_1 \rightarrow S_3$	105.2	4016.5	1.6×10^3
$T_1 \rightarrow S_4$	0.31	7268.6	1.3×10^{-10}
		Total rate	1.2×10^5

Section S3. Results of optimization of the (RO···OR') complexes on the singlet surface ^{1}(RO···OR').

Figure S2. Results of optimization of the (RO···OR') complexes on the singlet surface ^{1}(RO···OR'). Color coding: gray=C, white=H, red=O, blue=N.

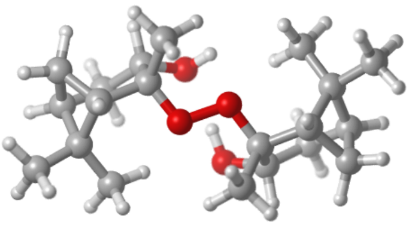

(*S*-alkoxy,*R*-hydroxy)$_2$

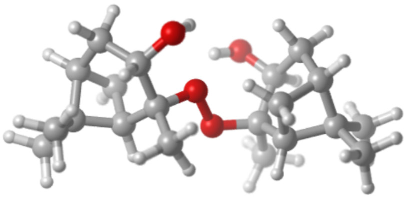

(*R*-alkoxy,*S*-hydroxy)$_2$

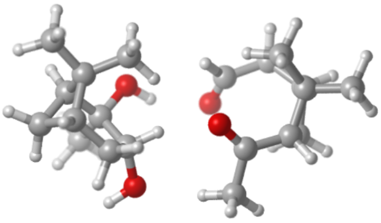

(*S*-alkoxy,*S*-hydroxy)$_2$

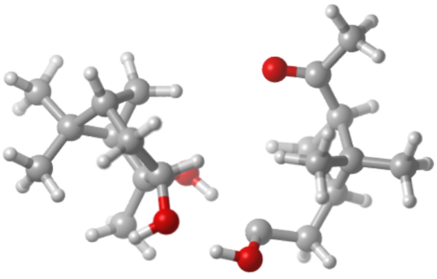

(*R*-alkoxy,*R*-hydroxy)$_2$

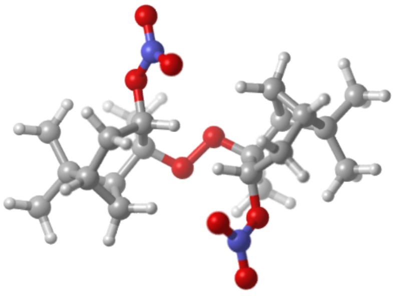

(*R*-alkoxy,*R*-nitroxy)$_2$

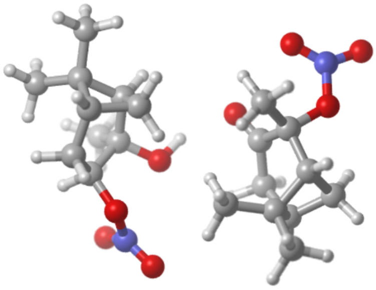

(*R*-alkoxy,*S*-nitroxy)$_2$

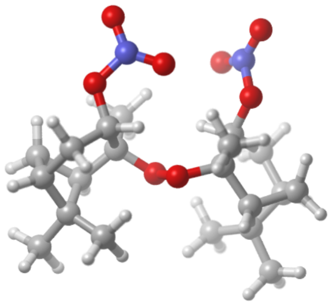

(*S*-alkoxy,*S*-nitroxy)$_2$

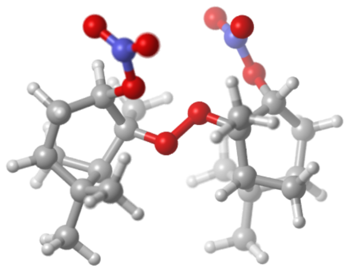

(*S*-alkoxy,*R*-nitroxy)$_2$

<u>**Section S4.** Optimized structures of the representative local minima conformer used for calculating the ISC rates in section S2 (even-numbered tables).</u>

Figure S3. Optimized structures and relative energies of the representative local minima conformers used to calculate the ISC rate in section S2. Color coding: gray=C, white=H, red=O, blue=N.

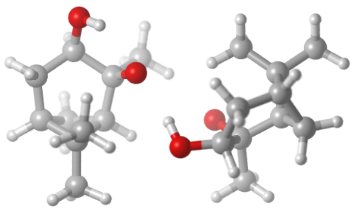

s

(*S*-alkoxy,*R*-hydroxy)$_2$
O-O distance = 3.45Å
$^a\Delta$E = 0.084 kcal/mol

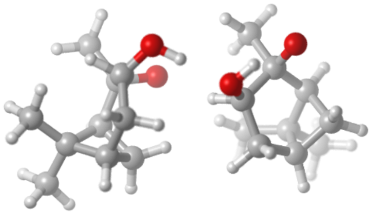

(*R*-alkoxy,*S*-hydroxy)$_2$
O-O distance = 4.87 Å
$^a\Delta$E = 1.862 kcal/mol

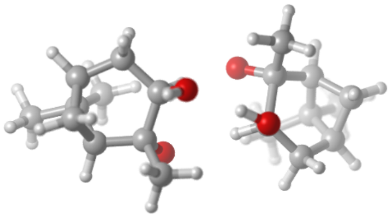

(*S*-alkoxy,*S*-hydroxy)$_2$
O-O distance = 3.6 Å
$^a\Delta$E = 1.021 kcal/ol

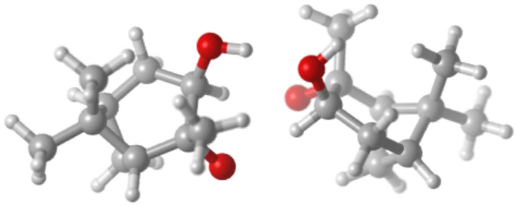

(*R*-alkoxy,*R*-hydroxy)$_2$
O-O distance = 3.5 Å
$^a\Delta$E = 0.013 kcal/mol

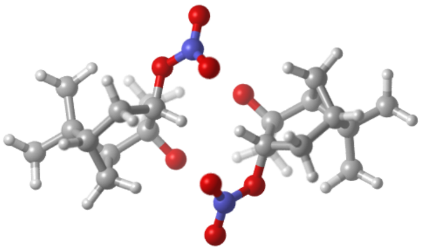

(*R*-alkoxy,*R*-nitroxy)$_2$
O-O distance = 3.2 Å
$^a\Delta$E= 0.0002 kcal/mol

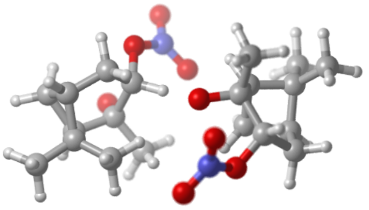

(*R*-alkoxy,*S*-nitroxy)$_2$
O-O distance = 5.48 Å
$^a\Delta$E= 0.388 kcal/mol

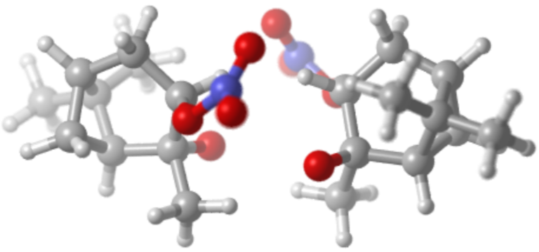

(*S*-alkoxy,*S*-nitroxy)$_2$
O-O distance = 3.97 Å
$^a\Delta$E= 0.012 kcal/mol

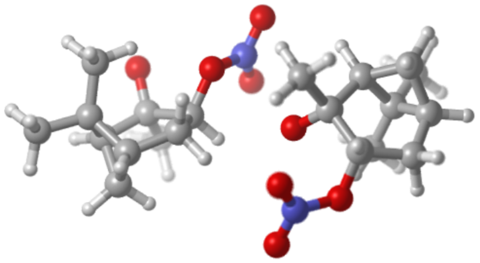

(*S*-alkoxy,*R*-nitroxy)$_2$
O-O distance = 5.21 Å
$^a\Delta$E= 0.182 kcal/mol

$^a\Delta$E= Difference in electronic energies relative to the global minima conformer in kcal/mol, at the ωB97X-D/6-31++G** level.
